# A 13-item Health of the Nation Outcome Scale (HoNOS-13): validation by item response theory (IRT) in patients with substance use disorder

**DOI:** 10.1186/s13722-023-00416-8

**Published:** 2023-10-24

**Authors:** Anne Chatton, Yasser Khazaal, Louise Penzenstadler

**Affiliations:** 1 Department of Psychiatry, Geneva University Hospitals, Geneva, Switzerland; 2 Addiction Medicine, Department of Psychiatry, Lausanne University Hospital and Lausanne University, Rue du Bugnon 23A, 1011 Lausanne, Switzerland; 3 Department of Psychiatry and Addictology, Montréal University, Montréal, Canada; 4 Division of Addiction Psychiatry, Department of Psychiatry, Geneva University Hospitals, Geneva, Switzerland; 5 Department of Medicine, University of Geneva, Geneva, Switzerland

**Keywords:** Substance use disorders, Symptom severity, HoNOS, Health of the Nation Outcome Scale, Item response theory

## Abstract

**Background:**

The Health of the Nation Outcome Scale (HoNOS) is a widely used 12-item tool to assess mental health and social functioning. The French version has an added 13th item measuring adherence to psychotropic medication. The aim of the current study is to uncover the unknown pattern of the new item 13 and to compare the unidimensional and multidimensional fit of the new HoNOS-13 using Item Response Theory (IRT). This research question was studied among inpatients with substance use disorder (SUD).

**Methods:**

Six hundred and nine valid questionnaires of HoNOS-13 were analyzed using unidimensional (one-factor) and multidimensional (two-factor) IRT modeling.

**Results:**

The multidimensional model suggesting a first factor capturing psychiatric/impairment-related issues and a second factor reflecting social-related issues yielded better goodness-of-fit values compared to the unidimensional solution. This resulted in an improvement of all slope parameters which in turn translates to better discriminative power. Significant improvement in item location parameters were observed as well. The new item 13 had a good discriminative power (1.17) and covered a wide range of the latent trait (− 0.14 to 2.64).

**Conclusions:**

We were able to validate the 13-item questionnaire including medication compliance and suggest that the HoNOS-13 can be recommended as a clinical evaluation tool to assess the problems and treatment needs for inpatients with SUD. Interestingly, the majority of item response categories are endorsed by respondents who are below and above the average levels of HoNOS. This indicates that the scale is able to discriminate between participants both at the low and at the high ends of the latent trait continuum. More importantly, the new item 13 has a good discriminative power and covers a broad range of the latent trait below and above the mean. It therefore has the desired profile of a good item and is a useful measure for the assessment of mental health and social functioning.

*Trial registration* ClinicalTrials.gov, Identifier: NCT03551301. Registered: 11.06.2018. Retrospectively registered, https://clinicaltrials.gov/ct2/show/NCT03551301.

**Supplementary Information:**

The online version contains supplementary material available at 10.1186/s13722-023-00416-8.

## Background

The Health of the Nation Outcome Scale (HoNOS) was developed by Wing et al. [1] as a brief general assessment of mental health and social functioning designed to measure a large range of problems of psychiatric patients and their evolution.

This first version was validated by exploratory factor analysis and gave rise to a 12-item scale evaluating four dimensions.^1^ Behavioral problems cover 3 items [1–3]: overactive, aggressive, disruptive or agitated behavior, non-accidental self-injury and problem drinking or drug taking. Impairment covers 2 items [4, 5]: cognitive problems and physical illness or disability problems. Symptomatic problems include 3 items [6–8]: problems with hallucinations and delusions, problems with depressed mood and other mental and behavioral problems. Social problems cover 4 items [9–12]: problems with relationships, problems with activities of daily living, problems with living conditions and problems with occupation and activities. Each item is scored 0 (no problems during the reporting period) to 4 (severe to very severe problem), higher categories reflecting more of the latent trait or greater severity. Analyses involving individual HoNOS items have been undertaken in many studies [3–5] (Additional file 1).

Since the launch of the first version, subsequent studies did not seem to reach agreement on the operationalization of HoNOS as the reproducibility of the above-cited dimensions found by Wing could not be demonstrated. Indeed, trying to replicate these findings and using a large sample of psychiatric patients, Trauer [6] found poor fit adjustment measures of the model to their data. Rather, they determined a five-scale model consisting of a ‘Depression’ scale (items 2, 7–9), an ‘Impairment’ scale (items 4 and 5), a behavior scale (items 1 and 3), a social problems scale (items 9–12) and a ‘Hallucinations/delusions’ scale (item 6) with item 9 cross-loading on Depression and Behavior factors. This structure was later replicated by Eagar et al. [7]. In a French validation study Lauzon et al. [8] found that the observed data fit neither the original four-factor structure nor an unidimensional model. In the same vein, several other factor structures including a unidimensional solution and a bifactor solution have been suggested but none of them have acceptable fit [9, 10]. For evaluation of populations in a community setting, a reduced unidimensional version of HoNOS-12 has been suggested [11, 12].

Despite these controversies, the HoNOS continues to be widely used to evaluate mental health patients in inpatient and ambulatory settings [13, 14].

Until now, the psychometric properties of HoNOS were measured for patients with general psychiatric disorders. Only few studies [15] have specifically measured these in patients with a main diagnosis of substance use disorders (SUD). In spite of several controversies related to HoNOS factorial structure, it was suggested that the items could help to identify sub-specific groups of patients with particular needs [16].

Confirmatory Factor Analysis (CFA) and Item Response theory (IRT) are two popular techniques for assessing the psychometric properties of a scale. Although both lead to the same conclusion, CFA assumes a linear relationship between the latent construct and the observed score at the item/subscale level whereas this relationship is non-linear under the IRT paradigm [17, 18]. IRT is a family of mathematical models^2^ parameterized under the logistic model for the analysis of binary, categorical and hybrid data (a mixture of the two). They are used to determine the parameters of an item based on the responses of individuals to that item [17]. Categorical IRT models include models for ordered and unordered data. When the items of a scale are polytomous-ordered (Likert-type) they are fitted in IRT by what is called the graded response model (GRM). This model, designed by Samejima [19], is one of the 2-PL IRT families. Thus, the HoNOS scale being polytomously scored makes it amenable to analysis by IRT-GRM.

Medication non-adherence is known to be an important factor influencing clinical outcomes [20]. This issue, mentioned in 2017 during a training session on HoNOS in Lausanne Switzerland [21] and named “problems with psychotropic medication compliance”, was first analyzed in 2018 as an added item to HoNOS-12 in a retrospective study comparing voluntary and involuntary admissions [22]. We think it is of utmost importance to formally take this 13th item into account in the overall therapeutic care of patients with SUD. To the best of our knowledge, the psychometric properties of the new HoNOS 13, consisting of the original items in HoNOS 12 plus the added one, have not been investigated yet.

Hence, using IRT-GRM, our aim is twofold:

1. to analyze HoNOS-13 as a unidimensional model (UIRT-GRM),
2. in the presence of lack of fit, to proceed with a two-factor model as an alternative multidimensional model (MIRT-GRM^3^).

## Methods

The data of this study were collected by experienced data extractors from the hospital electronic medical record system from February 2015 to September 2019. They concerned patients with SUD admitted to a specialized addiction unit of a large university hospital. The population were mainly men (70.7%), with a mean age of 43.3 (SD 11.5) years. During the reported period, the number of hospitalizations ranged from 1 to 13 with a median length of stay of 15 days (2–690). The median HoNOS score was 16 (1–44) at admission and 11 (0–37) at discharge. The questionnaire was administered by the psychiatrists working in the hospital unit who had received a training session for the use of this tool. The Geneva ethics comity approved this study (ClinicalTrials.gov, Identifier: NCT03551301). Six hundred nine (609) valid questionnaires of the HoNOS were analyzed.

### Statistical analysis

HoNOS is a polytomous-ordered categorical scale with its items ranked on a 5-point Likert scale from 0 (no problem) to 4 (severe to very severe problem), with higher scores indicating more problems. To handle this type of data, Samejima [19] proposed a probability function that a person’s response falls at or above a particular category given the latent trait as follows [23]:

$${P}_{jk}^{*}\left(\theta \right)=\frac{exp \left[{a}_{j}\left(\theta -{b}_{jk}\right)\right]}{1+exp \left[{a}_{j}\left(\theta -{b}_{jk}\right)\right]}.$$

This equation is known as the boundary characteristic function of item j for category *k*, given the latent trait θ. The parameter ${a}_{j}$ is the slope of the function or item discrimination and reflects an item ability to discriminate between individuals scoring high and low scores on θ. The ${b}_{jk}$ parameter also called threshold parameter refers to the latent trait where an individual has a 50% probability of endorsing a particular category *k* or higher.

Conceptually, GRM would treat each item as a series of $K-1$ dichotomous items, which translates into $K-1$ thresholds where $K$ is the number of Likert-type ordered categories [24].

In IRT, persons and items are located on the same continuum. A good differentiation among individuals i.e., the ability of an item at discriminating below and above the mean, is a desired characteristic of a good item [25].

The main concept in IRT is the item characteristic curve (ICC) produced by the model given in the above equation. They account for the relation between a person’s ability or trait and the probability of a particular item response.

Originally, a traditional IRT model contains a single continuous latent variable representing the construct of interest. The fitting of such a model requires the satisfaction of three fundamental assumptions: unidimensionality (the minimal assumption), monotonicity and local independence.

Unidimensionality means that item correlations are explained by a single dimension. This assumption was tested with the Loevinger’s H coefficients [26], which indicate the degree of homogeneity of an item set. Bounded by 0.3 and 0.4, H weakly supports unidimensionality. If bounded by 0.4 and 0.5, the scale is said moderately unidimensional. Higher values than 0.5 strongly satisfy the assumption of unidimensionality [27, 28]. The Mokken package of R program [29] was used for the calculation of the H values.

Monotonicity presumes a non-decreasing probability of endorsement of item response categories when the levels of the latent trait increase. This assumption was examined through the rest-score graphs as the difference between the raw scale score and the item score for each item. These graphs picture the rest-scores on the X-axis and the proportion of respondents in each rest-score group endorsing the item on the Y-axis [30]. The Mokken package of R program [29] was used to plot these graphs.

As for local independence, it assumes that the responses to an item are independent of that of the others, conditional on the person’s location [31–33]. This assumption is tested through the item residual correlation matrix. Residual pairs > 0.1 are an indication for local dependence [34, 35].

As psychological constructs became more complex, it also became obvious that the ability of a single construct to approximate complex data had reached its limits. Accordingly, psychometric research have led to the development of more sophisticated models of which MIRT is a novel statistical technique [36].

The 2-PL form of MIRT can be written as [37]:

$${P}_{jk}^{*}\left(\theta \right)=\frac{exp\left[{{\sum }_{m}a}_{jm}\left({\theta }_{m}-{b}_{jk}\right)\right]}{1+exp\left[-D{{\sum }_{m}a}_{jm}\left({\theta }_{m}-{b}_{jk}\right)\right]^{\prime }}$$

where ${P}_{jk}^{\text{*}}\left(\theta \right)$ is the probability that observed scores for item *j* and respondent *i* given the ability/trait θ to obtain a score greater than or equal to category *k*, ${a}_{jm}$ is the vector of item discrimination parameters for item *j* on each latent trait *m*, ${b}_{jk}$ is the vector of item severity parameters for each category k within item *j*, ${\theta }_{m}$ is the vector of the latent traits on the ${m}{\text{th}}$ dimension and *D* = 1 or 1.7, a scaling constant ( D = 1.7 to scale the logistic to the normal ogive metric, D = 1 to preserve the logistic metric).

Assumptions for using MIRT:

MIRT models differ from UIRT models in that they are a linear combination of a vector of abilities (θ) rather than a single dimension. Apart from that, the monotonicity and independence assumptions remain in force in MIRT models. The monotonicity assumption requires that as any element in the θ-vector increases, the probability of endorsing a certain item response category also increases. As for the independence assumption, it states that the response of any person to any test item is assumed to depend solely upon the person’s θ-vector and the item’s vector of parameters [38].

The model parameters were estimated using the Mirt package [39] of the free R program [29].

To recall, the Mirt package also allows for the estimation of unidimensional models by giving the program appropriate instructions.

Full information maximum likelihood estimation is implemented is this package for both unidimensional and multidimensional models.

A high discrimination parameter, resulting in a steep ICC, suggests that the item has a high ability to differentiate subjects with high and low levels of the construct [40]. A high discrimination also means that the item provides a lot of information on the latent trait. Nevertheless, items with low discrimination parameters, even though less informative, may contribute information over a wider spectrum of the latent trait. Descriptive rules of thumb guidelines for discrimination [41] suggest that: 0 = non discriminative power; 0.01–0.34 = very low; 0.35–0.64 = low; 0.65–1.34 = moderate; 1.35–1.69 = high; > 1.70 = very high; and + infinity = perfect.

Concerning the thresholds, there were five response options thus there are four of them. Table 1 pictures our sample distribution of HoNOS-13.

**Table 1:** Distribution of HoNOS-13

Item name	Item score	Response rate
1. Overactive, aggressive, disruptive or agitated behaviour	0	68.9
	1	15.2
	2	10.6
	3	3.8
	4	1.6
2. Non-accidental self-injury	0	82.1
	1	9.2
	2	5.3
	3	2.8
	4	0.6
3. Problem drinking or drug taking	0	12.0
	1	12.0
	2	19.7
	3	33.5
	4	22.8
4. Cognitive problems	0	72.9
	1	14.7
	2	8.5
	3	3.4
	4	0.6
5. Physical illness or disability problems	0	62.7
	1	16.5
	2	13.8
	3	5.9
	4	1.2
6. Problems with hallucinations and delusions	0	77.1
	1	8.9
	2	6.9
	3	4.6
	4	2.5
7. Problems with depressed mood	0	18.5
	1	23.8
	2	37.6
	3	15.6
	4	4.5
8. Other mental and behavioural problems	0	27.8
	1	18.3
	2	37.2
	3	13.5
	4	3.3
9. Problems with relationships	0	31.3
	1	31.5
	2	26.5
	3	8.5
	4	2.2
10. Problems with activities of daily living	0	38.7
	1	24.9
	2	24.4
	3	9.3
	4	2.7
11. Problems with living conditions	0	37.6
	1	22.8
	2	22.4
	3	11.5
	4	5.6
12. Problems with occupation and activities	0	20.8
	1	24.6
	2	35.3
	3	15.7
	4	3.7
13. Problems with psychotropic medication compliance	0	60.3
	1	12.9
	2	14.6
	3	7.5
	4	4.7

Using the data at admission, we first fitted a one-factor model for HoNOS-13 for the sake of parsimony and model complexity. Due to lack of fit, a two-factor model identified by two of the authors, psychiatrists (expert consensus) was envisaged: Factor 1 would capture psychiatric/impairment-related issues (items 1 to 8 and 13) and Factor 2 would reflect social-related issues (items 9 to 12).

Goodness of fit of the models was assessed by the root mean square error of approximation (RMSEA) of < 0.08 and < 0.06, respectively, and the comparative fit index (CFI) values of > 0.90 and > 0.95, respectively [42, 43]. Other information criteria, specifically the Akaike information criterion (AIC), Bayesian information criterion (BIC), and the sample-adjusted BIC (SABIC) were also used, knowing that AIC and BIC are specifically designed to penalize for model complexity.

Nested models were compared via the likelihood ratio statistics or by a reduction of goodness-of-fit indices such as AIC, BIC and SABIC. Finally, the performance of the UIRT and MIRT models was addressed through an anova testing which tests whether the more complex model is better at capturing the data than the simpler model.  A significant p-value (*p* < 0.05) speaks in favor of the more complex model.

All analyses, tests and plots were obtained using appropriate packages of the R program.

### Sample size requirements

Forero and Maydeu-Olivares [44] cited by Depaoli et al. [45] have found that sample sizes as small as 200 were sufficient for the parameter estimation of a graded response model. On the other hand, Jiang and al. also cited by Depaoli et al. [45] showed that a sample size of 500 provided accurate parameter estimates in the case a three-dimensional GRM composed from 30 to 90 items each with four response categories [46]. Thus, we are confident that the sample size at hand (609) fulfilled the necessary requirements for the analysis of a two-dimensional scale of 13 items with 5 response categories.

## Results

The GRM estimates for the UIRT model are presented in Table 2. This model also yielded goodness-of-fit statistic values of 0.896 for CFI and 0.0753 for RMSEA. These can be found in Table 3 (first line) as well as the other fit indices namely AIC, BIC and SABIC.

**Table 2:** Estimates for one-factor model (UIRT)

Item	Discrimination (slope)	Severity (threshold)			
Item no.	a	b1	b2	b3	b4
Item 1	0.61	0.71	2.17	4.49	6.99
Item 2	0.47	2.30	3.97	5.47	7.42
Item 3	1.01	− 3.33	− 2.83	− 2.03	− 0.02
Item 4	0.49	1.07	3.27	6.62	11.19
Item 5	0.29	0.62	2.84	6.88	12.40
Item 6	0.42	2.73	4.32	6.01	8.29
Item 7	0.64	− 3.28	− 1.70	0.87	3.70
Item 8	0.75	− 2.05	− 1.08	1.25	3.74
Item 9	1.89	− 1.07	− 0.12	0.95	2.25
Item 10	2.61	− 0.89	− 0.33	0.67	1.76
Item 11	1.69	− 0.93	− 0.19	0.79	1.89
Item 12	2.07	− 1.56	− 0.77	0.41	1.62
Item 13	0.97	− 0.16	0.54	1.70	3.03

**Table 3:** Comparison of model fit statistics and indices of the HoNOS-13

	Model	AIC	BIC	SABIC	RMSEA	CFI
HoNOS 13	UIRT (1—factor)	19389.4	19676.2	19469.8	0.0753	0.896
	MIRT (2—factor by expert consensus)	19327.0	19618.1	19408.6	0.067	0.919

The Loevinger’s coefficient, which informs on the degree of homogeneity of a scale and thus on its dimensionality, was *H = 0.22*, far below the minimum requirement.

Even though the unidimensionality assumption was not satisfied, we proceeded with the verification of that of local independence. We found that this assumption was not satisfied either as evidenced by the residual correlation matrix where several item pairs exceeded the 0.1 cut-off.

**Fig. 1:**
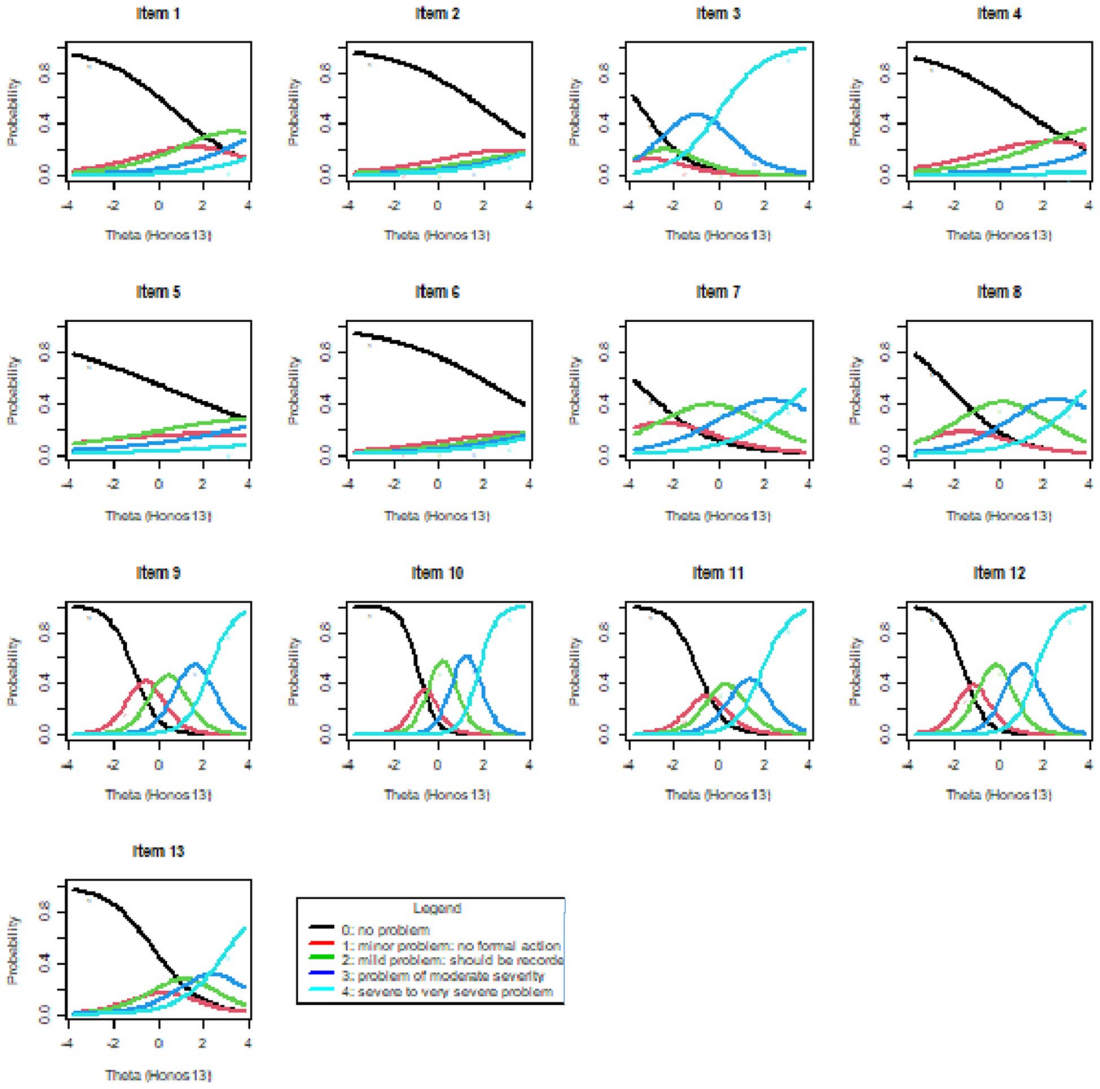
Item characteristic curves (ICC)

Finally, we present ICCs associated with the UIRT model to provide the reader a visual clue of the performance of each item (Fig. 1).

With respect to the MIRT model resulting by expert consensus, we obtained the following goodness-of-fit statistics: RMSEA = 0.067 and CFI = 0.919. These results together with the values of other fit indices: AIC, BIC and SABIC are depicted in Table 3, 2nd line. The fact that these indices were lower than in the unidimensional model and that the Anova test comparing the  performance of the two competing models yielded a significant result (p-value < 0.001) suggest that the MIRT model is superior to the UIRT one. With these empirical findings, we thus conclude that the 13-item scale can be conceptualized as a two-factor model and proceeded with the MIRT parameters estimation.

In Table 4 we present the GRM estimates for the MIRT model. In terms of the ranges proposed by Baker [41], we observed that items 9, 10, 11 and 12 had very high discriminative power with a range of 1.75–2.73, items 1, 2, 3, 4, 7, 8 and 13 had moderate discriminative power (range: 0.70 to 1.17) and items 5 and 6 showed very low to low discriminative power (range: 0.33 and 0.57). Items with positive thresholds only are said to discriminate above the mean (items 1 and 2, 4 to 6). Items 7 to 13 discriminate below and above the mean. It can be seen that item 3 (Problem drinking or drug taking) discriminates below the mean only. Considering the new item 13 (problems with psychotropic medication compliance) its thresholds (− 0.14, 0.47, 1.48 and 2.64) span a broad range of the latent trait below and above the mean. In terms of cumulative comparisons, a person with θ = − 0.14 has a 50% chance of answering 0 versus greater than or equal to 1, a person with θ = 0.47 has a 50% chance of answering 0 or 1 versus greater than or equal to 2, and so on.

**Table 4:** Parameter estimates for the MIRT model

Item no.	Discrimination (slope)		Severity (threshold)			
	a1	a2	b1	b2	b3	b4
Item 1	0.85		0.54	1.65	3.38	5.22
Item 2	0.77		1.51	2.59	3.54	4.76
Item 3	1.16		− 3.02	− 2.57	− 1.86	− 0.02
Item 4	0.70		0.77	2.35	4.71	7.89
Item 5	0.33		0.55	2.53	6.10	10.97
Item 6	0.57		2.11	3.33	4.61	6.33
Item 7	0.78		− 2.80	− 1.46	0.74	3.15
Item 8	0.99		− 1.65	− 0.87	1.01	2.99
Item 9		1.90	− 1.07	− 0.12	0.95	2.24
Item 10		2.73	− 0.88	− 0.33	0.66	1.74
Item 11		1.75	− 0.91	− 0.19	0.78	1.86
Item 12		2.16	− 1.54	− 0.76	0.41	1.59
Item 13	1.17		− 0.14	0.47	1.48	2.64

**Fig. 2:**
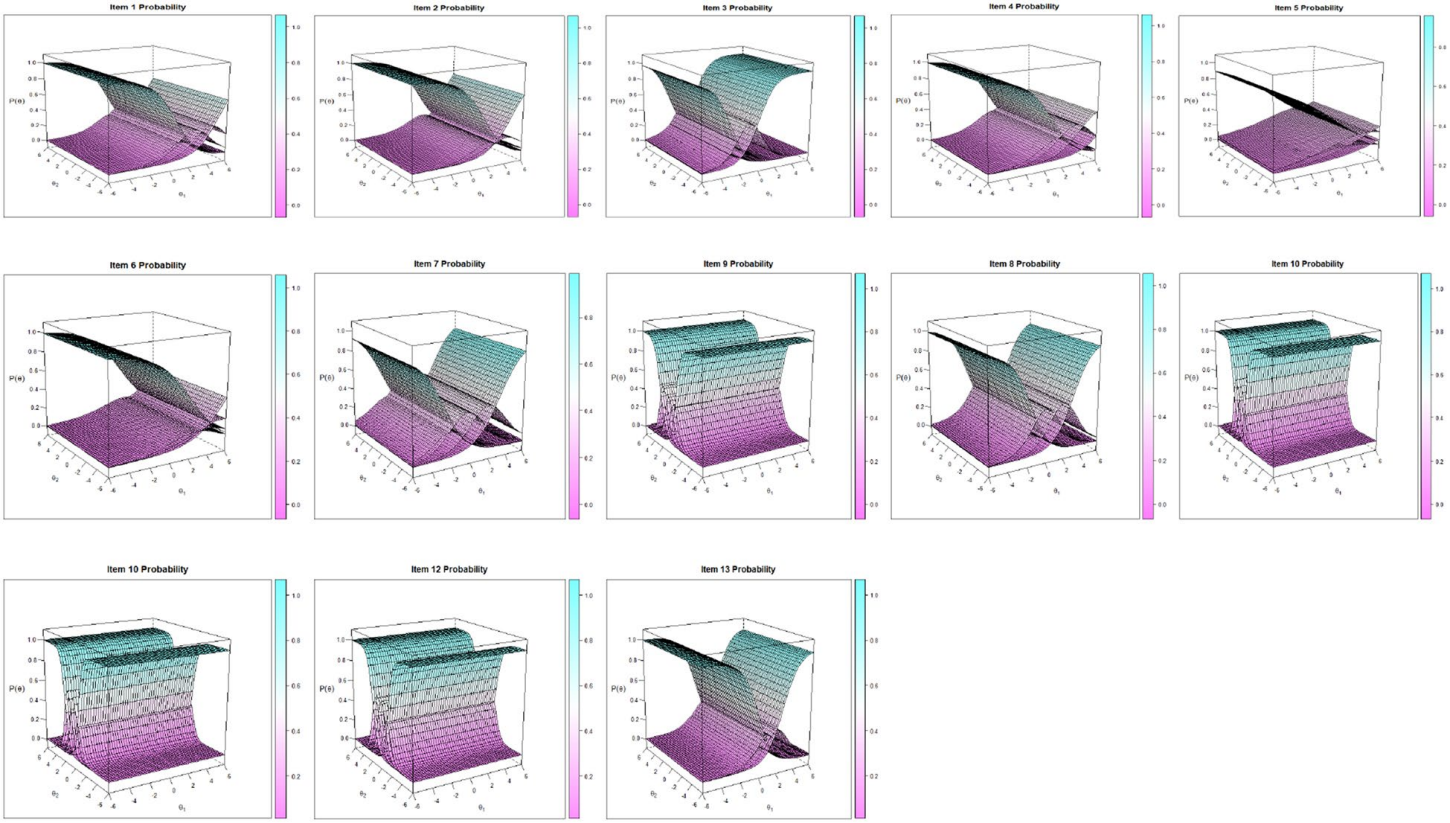
HoNOS 13 item characteristic surface

We present item characteristic surfaces (Fig. 2) as a visual tool to ensure their monotonic distribution.

**Fig. 3:**
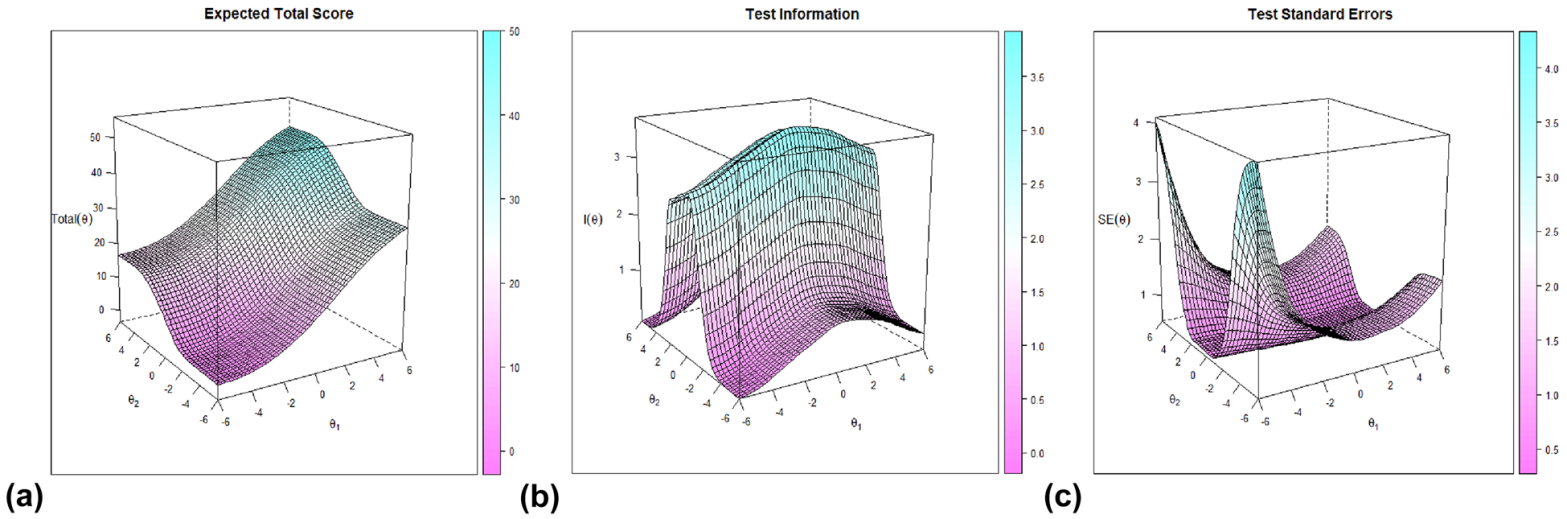
Expected total score surface, test information surface and test standard errors surface

Figure 3a shows the expected total scores as a surface plot which graphically quantifies the part of the latent trait space each person occupies. Different person location estimates can lead to the same expected trait score. Alternatively different person’s location will produce different trait scores conditional on a given factor.

The total information area index represents the area under the total information function (Fig. 3b). Because the items contribute independently to the total information function, the area under the total information function is the sum of all item information areas. In the multidimensional situation, as in the unidimensional case, there is a direct relationship between the slope of the ICCs and the amount of information. However, one difference resides in the fact that item information surface may be different for a point in the latent space depending on the direction used to cross the item response surface.

Finally, the test of the standard errors (SE) is a visual way to evaluate the precision of the latent trait estimates (Fig. 3c). To improve interpretability, SE is translated into the scale of reliability which assumes values between 0 and 1. The reliability of the first factor was 0.73 and that of the second factor 0.88.

## Discussion

The present study, the first to our knowledge, investigated the psychometric properties of the HoNOS-13 in a large sample of in-patients with SUD. The results do not support the existence of a one-dimensional instrument to be used as a primary outcome as attested by the weak Loevinger’s coefficient H value (*0.22*). Furthermore, while an acceptable value was obtained for RMSEA (0.0753), CFI (0.896) was not in the range of the expected cut-off. The multidimensional two-factor model of HoNOS-13 seems to reflect best the expert consensus approach. This model yielded better goodness-of-fit values compared to the one-factor solution and fulfilled the criteria of satisfactory RMSEA and CFI statistics (0.067 and 0.919 respectively). In addition, AIC, BIC and SABIC were lower than in the one-factor model. Finally, the highly significant p-value yielded by the Anova test (p < 0.001) strengthened our preference for the two-factor model. This model that groups psychiatric/impairment-related issues (symptoms) and social-related issues (problems) seems to confirm the hypothesis that the person’s response to an item is due to his or her location on the latent construct. Items 1 to 8 and 13 accounts more in the expected total score than the second one after standardization.

More importantly, the new item 13 has a moderate discrimination parameter (1.17) and covers a broad range of the latent trait. It is thus useful in the assessment of mental health and social functioning. This item may contribute in a more transdiagnostic way to the latent construct. Further studies using IRT on other populations are needed to assess the role of this item as well as valid external validation of the new scale. After a thorough literature review, we found a study [47] which includes a 13th item in the HoNOS. Using a psychogeriatric population aged 65+, they found that the scale was unidimensional. However, their results are derived from CTT analysis (Classical test theory). CTT is based on a different theory paradigm than IRT and has several shortcomings listed by Zanon et al. [23]. Furthermore, their added item called “drug management” may not have the same meaning or may not be understood the same way as ours called “medication adherence”. For all these reasons, their results do not allow for comparison with ours.

The negative thresholds of item 3 seem to indicate that this item discriminates more effectively respondents below the mean. This item, with large negative thresholds, seems to be endorsed by individuals with low levels of HoNOS. In reverse, items 1, 2, 4 to 6 are more effective for respondents above the mean. The lower loading observed for Factor 1 (especially for items 5 and 6) is likely due to the heterogeneity of the psychiatric symptoms [16, 48] assessed by the HoNOS. The higher loadings observed for the social-related issues may reflect a form of commonality of such problems among individuals with SUD and/or psychiatric disorders. Similar figures for the social-related items were observed in another study using a sample with psychiatric disorders [16].

We also found that the discrimination estimates for the items ranged from 0.33 to 2.73, indicating that some items of HoNOS-13, show rather low discrimination ability whereas others have high levels (Table 4). The strength of the factor loadings of items 5 and 6 in the two-component model is a matter of concern. However, item 5 measuring physical impairment and item 6 hallucinations seem to be less important in our specific group of patients with SUD. As the sample was taken from a specialized addiction unit, patients were typically treated for substance withdrawal and were less commonly admitted for acute psychiatric disorders. This may explain fewer problems with hallucinations (item 6) as found in a study by Andreas et al. [15]. Even though comorbid substance use is common among patients with psychotic disorders [49] these are more likely to be treated in psychiatric units. In the present sample, 22.9% of the subjects scored higher than zero in this item showing some kinds of symptoms, however not enough linked to overall severity of the latent trait (Table 4). A similar comment could be made for the items 5 (physical illness or disabilities problems) where 37.4% of the participants (scored from 1 to 4) on this item showing that such issues are common among patients with SUD [50, 51] however without having a strong contribution to catch the severity of the latent trait. Patients presenting important physical impairments are perhaps more often admitted to general hospital units for withdrawal and treatment of comorbid physical disorders. The removal of items 5 and 6 could yield stronger goodness-of-fit measures. But recalling that the development of a scale is not solely a question of statistical matter, model modification based on modification indices may result in models that lack external validity, highly susceptible to capitalization on chance. Therefore, the modifications should be defensible from a theoretical point of view [52]. For these reasons, a safe approach is to consider the scale in its integrality, that is, using all 13 items. Particularly removing such items could be problematic when considering other populations such as the ones admitted in acute psychiatric wards. However, the present data lead to expect loadings and IRT results variation according to the specific population (specially for the Factor 1, symptoms related items).

By contrast, the issues assessed by the Factor 2-related items were found to have very high discriminative power. These problems are common among patients with SUD as well as patients with other mental disorders [53, 54] and were also observed in studies using HoNOS in inpatients admitted for psychiatric disorders [16]. Importance of social problems among people with addictive disorders [55, 56], and their influence in the rate of service use [57] were repeatedly observed especially for more severe forms and longer duration of substance use. Social problems-related symptoms seem to play an important role in the overall severity. This highlights the importance of community and recovery-oriented interventions [58, 59] as well as for approaches focusing on transdiagnostic factors involved in such difficulties such as theory of mind [60] or self-stigma [61].

HoNOS-13 can be recommended as a clinical evaluation tool to assess the problems and treatment needs for inpatients with SUD. It is necessary to assess the two-factor model suggested in this study in other patient groups. It could be hypothesized that loadings and discriminative power may change across items depending on the clinical characteristics of a given population. For people with psychiatric and addictive disorders, the items related to the second factor and probably item 13 may show more constant characteristics.

This analysis presents one main limitation as it used routinely collected administrative and clinical data. It was therefore not possible to have more detailed information about individual patients such as specific measures on addiction severity, duration of treatment, and marital or family status. There was also no information concerning the type of addiction, however all patients were hospitalized for an addiction disorder as primary diagnosis. Another limitation is that our study could not demonstrate external validity with other studies of HoNOS with the same added item and using the same statistical paradigm. Thus, further studies using IRT on non-SUD populations are warranted. Indeed, the results of a study [47] of the HoNOS including a 13th item called “drug management” do not allow for comparison with our study for they derived from CTT analyses which are based on a different theory paradigm than IRT. Using a psychogeriatric population aged 65+, they found that HoNOS-13 was unidimensional. Also, their added item “drug management” may not have the same meaning or may not be understood the same way as “medication adherence”.

That said, in a sensitivity analysis we examined change in HoNOS over time using the data at discharge in the same setting. We were able to satisfactorily replicate the two-factor structure as evidenced by the following goodness of fit measures: RMSEA = 0.059, CFI = 0.945, AIC = 17371.7, BIC = 17662.8 and SABIC = 17453.3 (detailed estimates output not shown). These findings are to be linked with the reliability values of the data at entry reported early and those at discharge (Table 5). Hence, we are confident in the measurement reliability and stability overtime and acknowledge these results as a strength of the study.

**Table 5:** Two-factor model: reliability values

Factor description	Reliability	
	Data at entry	Data at discharge
Factor 1: psychiatric/impairment-related issues (items 1 to 8 and 13)	0.73	0.73
Factor 2: social-related issues (items 9 to 12)	0.88	0.94

The validation of HoNOS-13 in an adult population hospitalized for an addictive disorder shows that this tool can be used for these patients. As HoNOS is a widely used measurement in psychiatry, its validation for this population enables a shared reference point for comparison with general psychiatric patients. The findings demonstrate the validation of the two-factor model, encompassing psychiatric or impairment-related concerns and social-related issues. These factors help capture the severity of and monitor their clinical progress, thereby facilitating the organization of appropriate care. The validity of item 13, medication adherence, is important as it is known to directly influence clinical outcomes [20]. The individual items of HoNOS-13 allow clinicians to screen patients for social, psychiatric and treatment adherence and individual items can be discussed items in greater depth with patients if indicated.

## Conclusions

The 13-item questionnaire including medication compliance was validated in this analysis. Despite the above limitation, the HoNOS-13 including a question “Problems with psychotropic medication compliance” can be recommended as a valid clinical evaluation tool to assess the problems and treatment needs for inpatients with SUD. Interestingly, the majority of item response categories are endorsed by respondents who are below and above the average levels of HoNOS. This indicates that the scale can discriminate between participants both at the low and at the high ends of the latent trait continuum. More importantly, the new item 13, with a moderate discrimination parameter and covering a broad range of the latent trait has the desired profile of a good item. It is thus useful in the assessment of mental health and social functioning.

### Supplementary Information

**Additional file 1:** Honos Items of all participants.

## Data Availability

Data is available in a Additional file 1.

## Abbreviations

AIC: Akaike information criterion
BIC: Bayesian information criterion
CFA: Confirmatory Factor Analysis
CFI: Comparative fit index
CTT: Classical test theory
GRM: Graded response model
HoNOS: Health of the Nation Outcome Scale
ICC: Item characteristic curve
IRT: Item response theory
MIRT: Multidimensional extension of the IRT
RMSEA: Root mean square error of approximation
SABIC: Sample size adjusted BIC
SUD: Substance use disorder

## Footnotes

1 The terms “dimension” and “factor” as distinct parts of a latent construct are used interchangeably throughout the remainder of this article.

2 1-PL (1-parameter logistic), 2-PL and 3-PL are the IRT family models. In each of them, 1, 2 respectively 3 parameter(s) are to be estimated.

3 As this article focuses on IRT graded models, we will drop the term “GRM” in UIRT-GRM and MIRT-GRM and use UIRT and MIRT in the rest of this document for the sake of simplicity in both writing and reading.
